# RT-LAMP assay for rapid detection of the R203M mutation in SARS-CoV-2 Delta variant

**DOI:** 10.1080/22221751.2022.2054368

**Published:** 2022-03-30

**Authors:** Jianing Yang, Xuejiao Hu, Wenzhuo Wang, Yujing Yang, Xinqiang Zhang, Wei Fang, Lei Zhang, Shan Li, Bing Gu

**Affiliations:** a MOE International Joint Laboratory for Synthetic Biology and Medicines, School of Biology and Biological Engineering, South China University of Technology, Guangzhou 510006, P.R. China; b Laboratory Medicine, Guangdong Provincial People’s Hospital, Guangdong Academy of Medical Sciences Guangzhou 510000, P.R. China; c NMPA Key Laboratory for Quality Control of Blood Products, Guangdong Institute for Drug Control, Guangzhou 510663, P.R. China

**Keywords:** SARS-CoV-2, Delta variant, R203M mutation, RT-LAMP, rapid diagnosis

## Abstract

The highly infectious Delta variant strain of SARS-CoV-2 remains globally dominant and undermines COVID-19 vaccines. Rapid detection of the Delta variant is crucial for the identification and quarantine of infected individuals. In this study, our aim was to design and validate a genotyping RT-LAMP method to detect Delta variants specifically. R203M in the *N* gene of SARS-CoV-2 was chosen as the Delta variant-specific mutation for genotyping. To target the R203M-harboring region and the conserved sequence of the *N* gene, two sets of primers were designed, and a Cq (quantification cycle) ratio-based RT-LAMP for SARS-CoV-2 and R203M detection was developed by analyzing the significant discrepancy in amplification efficiency of the two sets of primers. We validated the RT-LAMP method on 498 clinical specimens in parallel with RT-qPCR, and 84 Delta variants from 198 positive samples were determined by sequencing. Compared with traditional RT-qPCR analyses, RT-LAMP appears to be 100% accurate in detecting SARS-CoV-2 clinical samples. RT-LAMP has a good ability to distinguish between Delta and non-Delta variants under a Cq ratio threshold of 1.80. Furthermore, the AUC (area under the curve) of this method was 1.00; the sensitivity, specificity and accuracy were all 100%. In summary, we have proposed a rapid, accurate and cost-effective RT-LAMP method to detect SARS-CoV-2 and Delta variants, which may facilitate the surveillance of COVID-19.

## Introduction

The Delta variant strain of SARS-CoV-2 (also known as the B.1.617.2 and AY lineages) was first noted in India in October 2020 and has since become the most widely spread and dominant strain worldwide. Infection with Delta is characterized by a significant increase in infectiousness, viral load, and risk of disease progression compared with those of the wild-type strains, which has contributed to a resurgence in countries that had previously been able to suppress COVID-19 outbreaks and even in those with highly vaccinated populations [1–4]. Recent studies have demonstrated that patients infected with Delta variant have a 1000-fold higher viral load than lineage A/B when oropharyngeal swabs first become PCR-positive [2], and patients infected with Delta have more severe symptoms and higher mortality [5]. The immune effect of a single vaccine injection against Delta is as low as 33%, with 60%−88% of the immune effect for the two injections. Several types of vaccines were tested, including the BNT162b2 vaccine (Pfizer-BioNTech) and the ChAdOx1 nCoV-19 vaccine (AstraZeneca) [3,4].

The timely diagnosis and monitoring of the mutant strains are crucial to providing appropriate treatment for COVID-19 patients and to implementing public health strategies to counter these and future strains [6]. The gold standard for detecting emerging SARS-CoV-2 variants is viral genome sequencing; however, sequencing is generally infrequent, because it requires sophisticated equipment and highly trained personnel which is expensive and time-consuming. RT–PCR has become widespread for SARS-CoV-2 detection by laboratories, but routine RT-qPCR cannot distinguish variants or can only by produce negative results or Tm-shift melting curves according to the reported RT–PCR studies [7,8], which may lead to equivocal results and still require sequencing confirmation [9]. Researchers are increasingly realizing that there is an urgent need for more rapid, simple and accurate SARS-CoV-2 mutation identification strategies to meet the current challenges from the emergence and spread of the Delta epidemic. The exploration of the RT-LAMP (reverse transcription loop-mediated isothermal amplification) protocol, with its nature of easy use and extensibility, rapid amplification of nucleic acids and high specificity, represents an attractive approach to this type of assay.

LAMP uses 4–6 primers to identify 6–8 specific regions on the target gene, and it can achieve efficient amplification at a constant temperature. LAMP amplification is primarily performed using inner primers and outer primers, and the addition of loop primers has greatly improved the reaction rate [10,11]. The RT-LAMP assay could be used as an alternative point-of-care test to detect SARS-CoV-2 with sensitivity in the field and/or resource-limited settings [12–17]. Apart from LAMP, RPA is another isothermal amplification technology that is also a common method used to detect SARS-CoV-2. Compared with LAMP at 65 °C for 40 min, RPA can provide amplification results within 30 min at 37 °C, and in principle, only one pair of primers [18,19] is needed. However, the reaction system of RPA requires the participation of multiple enzymes [20], and it is more complex and less mature than the LAMP system. In addition, LAMP currently has a variety of derivative methods for use toward SNP detection. For example, the PE-LAMP (probe enhanced loop-mediated isothermal amplification) method has been successfully applied to single-base genotyping [21]. The mechanism of SNP detection is based on the difference in amplification rate using wild-type and mutant templates. The mutant may be detected by tracking the time gap between these two reactions. In this study, we hope to develop a visual RT-LAMP assay of novel real-time fluorescence for the rapid identification of Delta variants based on their unique mutations.

Delta variants have a number of characteristic mutations in their spike protein, including single-nucleotide polymorphisms (SNPs) resulting in T19R, R158G, L452R, T478 K, D614G, P681R, and D950N, which are usually chosen as the detection targets of the molecular diagnostics for Delta genotyping [22,23]. Despite these highly prevalent mutations within the *S* gene, researchers found that only approximately 6.3% of these mutations in the S protein are specific, with the remainder distributed across the SARS-CoV-2 proteome. Nucleocapsid (*N*) protein is a major contributor to RNA packaging and viral replication and is the most commonly recommended target for SARS-CoV-2 detection; however, mutations within the *N* gene have not been well documented. Newly reported *N* mutations, such as R203M and D377Y, are highly exclusive to the Delta variant [23]. The development of rapid molecular testing for Delta-specific mutations of interest will enable laboratories to monitor variants promptly and expediently and therefore implement more targeted and appropriate surveillance and control programs.

A novel real-time fluorescent visualization RT-LAMP assay based on the R203M mutation was developed and validated, and this strategy allowed for the discrimination of Delta variants while detecting SARS-CoV-2 simultaneously.

## Materials and methods

### Target selection and plasmid preparation

We tracked the SARS-CoV-2 genome sequences from the GISAID database and analyzed real-time mutation situation reports from the standardized, open-source database of COVID-19 (https://outbreak.info/). Target mutation sites were selected from typical mutations in the Delta variant, and the sequence region containing the mutation was analyzed by BLASTN to ensure that the region used for designing the RT-LAMP primers was specific to Delta. In addition, the conservative fragments that did not contain any mutations in the *N* gene were selected as the “internal reference” template.

The wild-type SARS-CoV-2 *N* gene plasmid (GenBank MN908947.3, 28274∼29533nt) was synthesized by Tsingke Biotechnology. The synthetic wild-type *N* gene plasmid was used as a wild-type positive control in the RT-LAMP reaction. Site-directed mutagenesis was performed on the 608th nucleotide of the wild-type *N* gene plasmid, which is the R203M mutation (G→T). Similarly, R203K/G204R (GGG→AAC) and T205I (C→T) mutations were also prepared. Then, the mutant plasmid was purified with a plasmid purification kit (TIANGEN BIOTECH), and sequenced by Tsingke Biotechnology. The quality and concentration of DNA were determined with a Nanodrop UV-Vis spectrophotometer, and the nucleotide copy number was calculated accordingly.

### Sample collection and RNA extraction

A total of 498 nasopharyngeal swab samples were collected from Guangdong Provincial People's Hospital and the Centers for Disease Control and Prevention of Guangdong Province. The swabs were preserved in 2 mL of virus preservation solution (TianLong), which inactivated the viruses and preserved the samples. The total RNA was extracted from the nasopharyngeal swabs by using a QIAamp Viral RNA mini kit (QIAGEN) in a biosafety level 2 laboratory. The RNA extracted from 498 nasopharyngeal samples was subjected to the RT–PCR and RT-LAMP assays in a double-blind manner parallelly.

### Primer design

**Figure 1:**
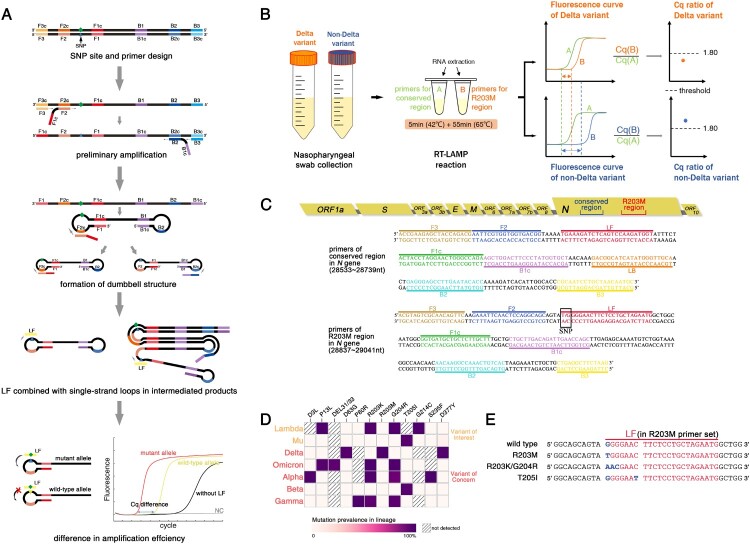
. RT-LAMP strategy for SARS-CoV-2 Delta and selection of target and primers. (A) The principle of detecting SNPs using RT-LAMP. SNP genotyping could be performed by tracking the time gap between these two reactions, which could be visually reported using the quantitative fluorescent Cq-difference. (B) The pipeline of the Cq ratio-based RT-LAMP method in detecting Delta. The extracted RNA from clinical samples was amplified with two sets of primers (R203M and conserved primer). (C) Schematic view of two sets of RT-LAMP primer target sites. The R203M mutation (G to T transition) in the mutant primer is indicated in the black box, G refers to the wild allele and T is the mutant allele in the Delta (sequence from GenBank: MN908947.3). (D) Heat map of the observed mutations within the N protein as of February 2022. Each row corresponds to the variant of SARS-CoV-2 listed on the left; each column indicates mutations listed on the top. (E) The specific differences in the R203M, R203K/G204R and T205I mutations in the *N* gene sequence.

Candidate amplification primers, including outer primers (F3/B3), inner primers (FIP/BIP), and loop primers (LF/LB) were designed using the Primer Explorer V5. During the RT-LAMP detection of Delta R203M, a single loop primer (LF or LB) close to the R203M-containing region was designed instead of a pair of loop primers (both LF and LB) to achieve distinct single nucleotide mutation discrimination. The RT-LAMP primers in the conserved region of the *N* gene were designed according to common principles, that is, six complete primers. All the primers were synthesized by Guangzhou Tianyihuiyuan Biotechnology and are shown in Figure 1C and Table 1, respectively.

**Table 1.** Primer sequences of the developed RT-LAMP assay

Primer Set	Primer	Sequence(5’to3’)
R203M primer set	PE1-F3	ACGTAGTCGCAACAGTTC
	PE1-B3	CTTAGAAGCCTCAGCAGC
	PE1-FIP	AAGCAAGAGCAGCATCACCG-GAAATTCAACTCCAGGCAGC
	PE1-BIP	CTGCTTGACAGATTGAACCAGC-GTGACAGTTTGGCCTTGTT
	PE1-LF4	CATTCTAGCAGGAGAAGTTCCC
Conserved primer set	A1-F3	ACCGAAGAGCTACCAGACG
	A1-B3	GCATTGTTAGCAGGATTGCG
	A1-FIP	TCTGGCCCAGTTCCTAGGTAGT-AATTCGTGGTGGTGACGG
	A1-BIP	AGCTGGACTTCCCTATGGTGCT-GGTGTATTCAAGGCTCCCTC
	A1-LF	ACCATCTTGGACTGAGATCTTTCA
	A1-LB	GACGGCATCATATGGGTTGCA

*^a^*: The “A” with black border in PE1-LF4 is complementary to R203M mutant T allele.

### RT-LAMP reaction

The RT-LAMP amplification system was set to 25 μL per reaction, which contained 10× Bst Buffer (200 mM Tris-HCl, 100 mM (NH_4_)_2_SO_4_, 100 mM KCl, 20 mM MgSO_4_, and 1% Triton-100, pH 8.8), 1.6 mM dNTPs (Sangon Biotech), 0.8 M betaine (SIGMA), 25× primer mix (40 mM inner primer, 20 mM loop primer, and 10 mM outer primer), 8 mM MgSO_4_ (SIGMA), 8 U Bst DNA polymerase (Optimized internally[24]), 5 U Reverse Transcriptase AMV (Takara Biotechnology), 1/25,000 diluted original SYTO 9 Green-Fluorescent Nucleic Acid Stains (Invitrogen Thermo Fisher Scientific), and 5 μL of nucleic acid template. The final volume was brought up to 25 μL with DEPC water. Real-time fluorescent RT-LAMP reactions were performed for 5 min of incubation at 40 °C for reverse transcription, followed by 65°C for 55 min for the amplification in a QuantStudio 12 K Flex Real-Time PCR System (Applied Biosystems). The fluorescence measurement value was recorded every minute during 65°C constant temperature amplification, and the fluorescence detection channel was 470/514 nm (for SYTO9).

### RT-LAMP primer specificity analyses

The primer specificity was evaluated using a BLAST alignment of primer sequences to those of the *N* gene from SARS-CoV (GenBank DQ182595.1), MERS-CoV (GenBank NC019843.3), Bat SARS CoV HKU3 (GenBank DQ022305.2), H1N1 (GenBank NC026436.1), Influenza B (GenBank NC-002208.1), HPIV-1 (GenBank NC003461.1), and HRV-A66 (GenBank MN749158.1). The primer specificity was further measured through cross-reactivity analyses with other pathogens. DNA or RNA templates of 12 common viral pathogens, namely SARS-CoV, MERS-CoV, influenza B virus, H1N1/H3N2 influenza A virus, MP, EV-U/71, AdV-B/E, HPIV-1/2/3, HCMV, CA16, RSV and CPN were amplified via RT-LAMP. Five microliters of the pathogen's RNA or DNA was used as a template in each test. The negative control used the same amount of DEPC water instead of nucleic acid as a template, and four plasmids for the *N* gene (wild-type, R203M, R203K/G204R and T205I mutations) at a concentration of 10^8^ copies/mL were used as positive controls.

### Lod of the RT-LAMP assay

To determine the detection limit (LOD) of RT-LAMP for Delta and non-Delta strains, samples from two types of SARS-CoV-2 RNA (wild-type and R203M-mutant) in 2-fold serial dilutions were used as the template in RT-LAMP reactions with the R203M primer set. Wild-type SARS-CoV-2 RNA was also serially diluted and tested in RT-LAMP reactions with the conserved primer set to obtain the LOD for the conserved region of the SARS-CoV-2 *N* gene. A quantification cycle value of less than 55 Cq (Supplemental Figure3) was defined as a positive result, and the minimum concentration of the positive reaction was recorded. Samples of all concentrations were subjected to 10 replicates to estimate a 95% detection limit using a probit regression model.

### The Cq ratio-based RT-LAMP method

The Cq ratio-based RT-LAMP method was established to detect the R203M mutation. The wild-type and R203M *N* gene plasmids (from 10^3^ to 10^8^ copies/mL) were used as templates, the wild-type and R203M primers were used, and three replicates were performed for each concentration. Ratio calculations and analysis were performed on the Cq values. RNA from clinical specimens was then used as a template to calculate the Cq ratio, including the wild-type, Delta and non-Delta variants (20A, 20I (Alpha, V1), and 20H (Beta, V2)), which cover common sequence types near the R203M mutation site.

### RT-qPCR

An authorized commercial RT-qPCR kit (Da'an Gene) was used to analyze the clinical swab samples, which could be used to appraise the *N* and *ORF1a/b* gene of SARS-CoV-2. Each 25 μL RT-qPCR sample consisted of 17 μL of reaction buffer, 3 μL of enzyme mix, and 5 μL of template RNA. The fluorescence channels were FAM for the *N* gene and VIC for the *ORF1a/b* gene. A quantification cycle value of less than 30 was defined as a positive test based on the given instructions.

### Next-generation sequencing

The SARS-CoV-2-positive samples were further analyzed with an ULSEN® Kit for NGS target enrichment of the whole SARS-CoV-2 genome (MicroFuture) and sequenced on an Illumina NovaSeq paired-end 150 platform to determine whether they were Delta or non-Delta, according to the manufacturer’s protocol. The types of variant strains are recorded in Supplemental Table 3.

## Statistical analysis

The clinical sensitivity, specificity, and predictive values were calculated using OpenEpi V3 software (http://www.openepi.com/DiagnosticTest/DiagnosticTest.htm) for the RT-LAMP and RT-qPCR assays.

### Ethics statement

All the participants in the study written informed consent, and the ethics committee of each participating institution approved the study. The samples used here were collected during COVID-19 tests and were not an extra burden on the patients.

## Results

### Delta-specific mutation selection

The Delta variants contain B.1.617.2 and all AY.* lineages according to analysis of all the SARS-CoV-2 sequences from the GISAID and WHO websites. Since the SARS-CoV-2 N protein plays a central role in increasing the replication of natural variants, and the *N* gene is one of the officially recommended targets for nucleic acid detection, we therefore analyzed the Delta mutations located in *N* gene. We selected a unique Delta mutation based on the criteria of a mutation rate >90% in all Delta sequences. According to the Delta mutation heat map (Figure 1D), R203M, D63G, and D377Y mutations were found in 98.6%, 96.9% and 97.8% of Delta variants, respectively, which rarely occurred in other VOC or VOI variants (≤ 0.1%).

The position of the SNP should also be considered for primer design; R203M (G→T, 28881nt) was located in the middle of the *N* gene sequence, which was conducive to primer design and screening for RT-LAMP of SNP detection, whereas the terminally located D63G and D377Y (28461nt and 29402nt) inherently restricted the primer design and selection for this developed method. However, near the R203M mutation site, the other common mutations in VOC and VOI were primarily R203K/G204R and T205I, and their base changes in the gene sequence are shown in Figure 1E. The difference between the R203K/G204R, T205I, and Delta variants are more noticeable when compared to the wild-type *N* gene. The actual detection was also beneficial for the resolution of this mutation and the identification of Delta by the R203M LAMP primer. Taken together, R203M of the *N* gene was chosen as a well-positioned candidate to distinguish Delta from other SARS-CoV-2 variants in the subsequent RT-LAMP experiment.

### Construction of the Cq ratio-based RT-LAMP method

#### R203M primer design and screening

**Figure 2.**
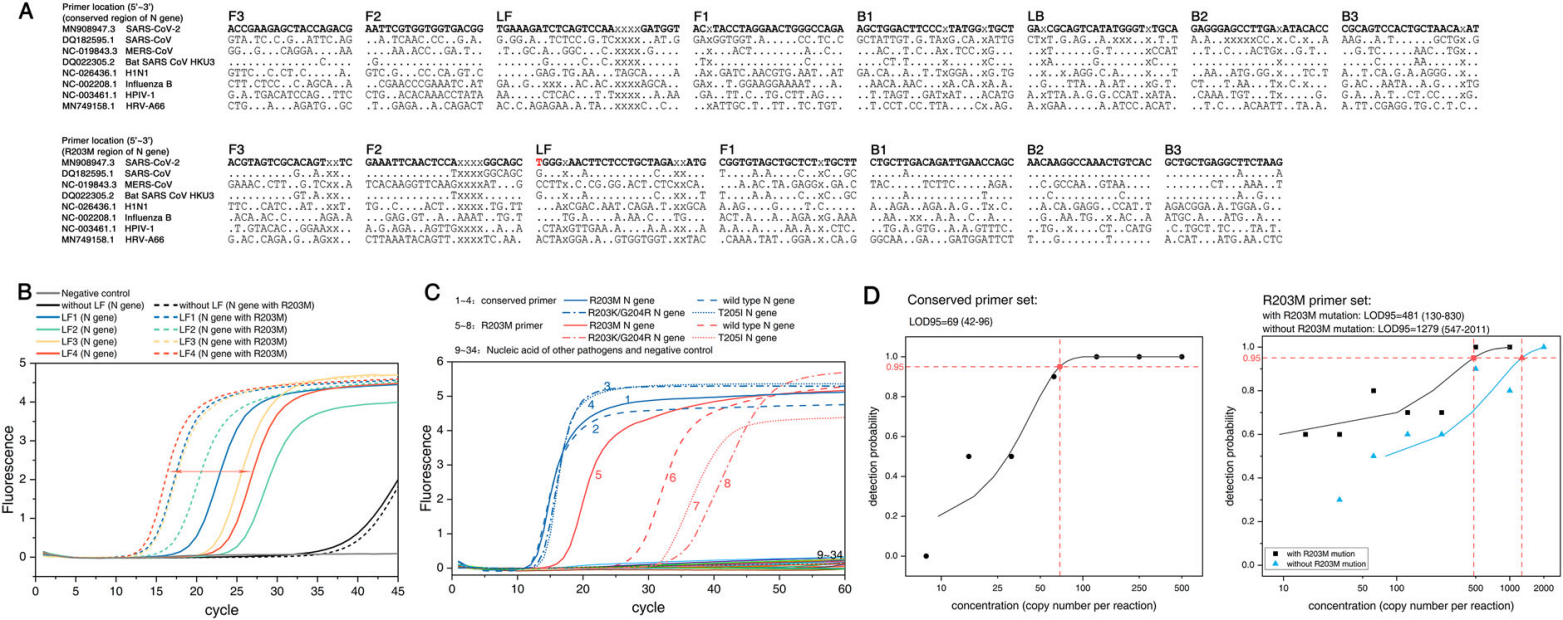
R203M primer screening, and the analytical specificity and detection limit of the two sets of RT-LAMP primers. (A) Primer sequence comparisons for six viruses (SARS-CoV, MERS-CoV, Bat SARA CoV HKU3, H1N1, Influenza B, HPIV-1 and HRV-A66) The red “T” in the LF of the R203M primer set is the mutation site of R203M. (B) Selection of loop primers in the mutant primer set. The selected LF4 shows the best distinguishing effect for R203M. (C) The analytical specificity analyses of RT-LAMP. Numbers 1–4: the amplified curve of the R203M, R203K/G204R, T205I mutant-containing template and wild template using the conserved primer set. Numbers 5–8: the amplified curve of the R203M, R203K/G204R, T205I mutant-containing template and wild template using the R203M primer set. Numbers 9–20 indicate RT-LAMP reactions using the conserved primer set for detecting 12 pathogenic plasmids consecutively (SARS-CoV, MERS-CoV, H1N1/H3N2 of influenza A viruses, influenza B viruses, MP, EV-U/71, HPIV-1/2/3, AdV-B/E, CA16, Cpn, RSV and HCMV). Numbers 21–32 refer to RT-LAMP reactions using the R203M primer set for these 12 pathogens. Numbers 33 and 34 were DEPC H_2_O (negative control) with the conserved primer set and the R203M primer set, respectively. The RT-LAMP assay did not cross-react with other human-pathogenic coronaviruses and common viral pathogens. (D) The 95% limits of detection (LOD95) of the RT-LAMP. The nucleic acid concentration was determined from 2000 copies/reaction to 7.83 copies/reaction. The LOD95 of the conserved region primer set (left) and the LOD95 of the R203M primer set (right). A probit regression model was used to estimate the LOD95. A cycle threshold value of less than 55 was defined as positive.

We designed R203M allele loop primers and their respective inner and outer primers based on the LAMP principle (Figure 1A). Due to the difference in amplification efficiency between the two reactions, the R203M genotyping could be performed using the time gap, which could be visually reported in a fluorescent Cq-difference way. Six primer sets were initially designed, each consisting of two outer primers (F3/B3), two inner primers (FIP/BIP) and a single loop primer (LF or LB) that corresponds to the allelic type of R203M (Supplemental Table 1). It is critical to select the LP to obtain the ideal discrimination, and the R203M discrimination ability of these primer sets was subsequently tested with RT-LAMP reactions using both wild-type and R203M mutant-type plasmids at the same template concentration of 10^8^ copies/mL (Figure 2B, Supplemental Table 1 and Supplemental Figure 1). One RT-LAMP primer set was ultimately selected as the optimal primer (R203M primer, for short, Table 1, Figure 1C, Figure 2B), which could provide a minimum Cq-gap of 10 min (the amplification conditions were set to 1 min per cycle).

#### Conserved primer design and screening

The RT-LAMP for the Delta mutation was calculated based on the concentration of the target, and crudely treated or unknown samples may challenge the application of this method in clinical scenarios. We therefore proposed a Cq ratio-based RT-LAMP procedure to differentiate Delta from other SARS-CoV-2 strains qualitatively. The principle underlying our strategy is shown in Figure 1B. A conserved region in the *N* gene of SARS-CoV-2 was introduced as the “internal control”, and a corresponding RT-LAMP assay targeting this conserved segment was developed to indicate the concentration of the samples. Because the SNP allele sits in the loop domain of the RT-LAMP products, the G to T transition of R203M could lead to an amplified Cq change for the R203M mutation while barely affecting the Cq value of the conserved fragment of the *N* gene; R203M consequently alters the ratio of the Cq value for the mutant-harboring fragment to the Cq for the conserved sequence of the *N* gene.

We analyzed all the mutations of the *N* gene and found two conserved regions (28514–28833nt and 29044–29393nt) (minimum conservation at any nucleotide position was 99%). We designed four sets of RT-LAMP primers targeting the two regions (Supplemental Table 2), and one set of primers targeting the conserved 28514–28833nt presented the best amplification efficiency, which was selected as the “internal reference” primer (called the “conserved primer” for short, Table 1, Figure 1C, Supplemental Table 2 and Supplemental Figure 2).

#### The Cq ratio-based RT-LAMP method

**Figure 3.**
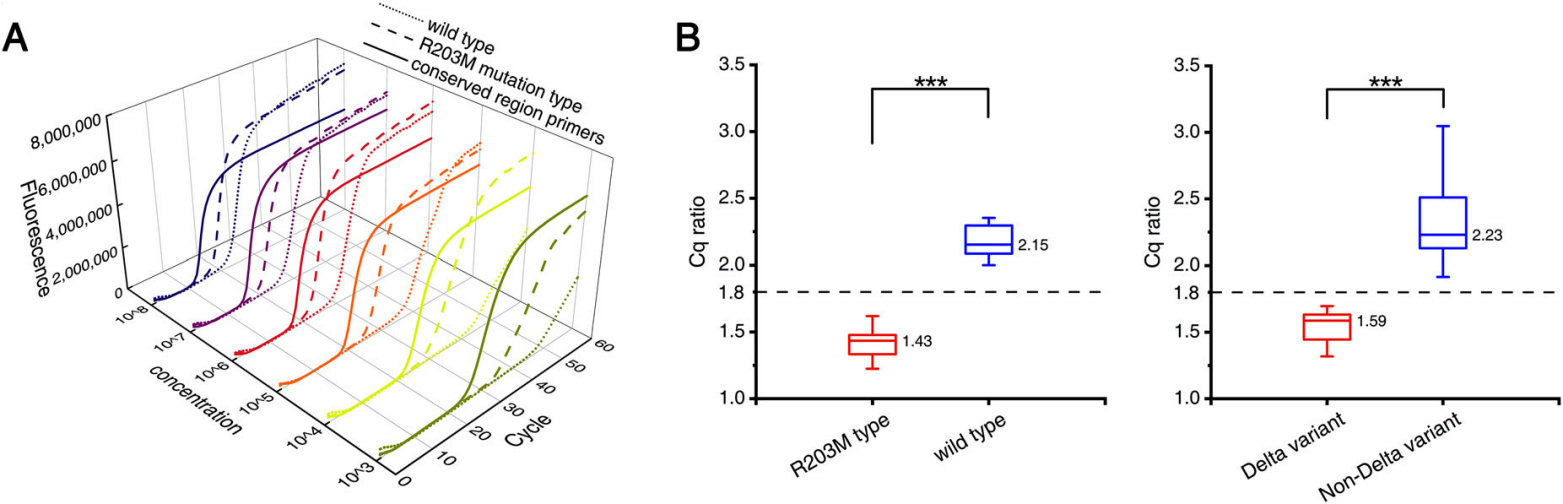
The Cq ratio-based RT-LAMP method. (A) The real-time quantitative fluorescent RT-LAMP result of the conserved and R203M primers over a range of templates from 10^3^ −10^8^ copies/mL. The amplification curves: wild-type plasmid using the conserved primers (solid lines), wild-type plasmid using the mutant primers (dotted lines), and R203M mutant plasmid using mutant primers (dashed lines). (B) The Cq ratio of the mutant plasmid and wild-type plasmid (left). The Cq ratio of the Delta variant and non-Delta variant (right). Box plots indicate the minimum and maximum value (whiskers), median (middle line), and 25th and 75th percentiles (box). The cutoff of the Cq ratio is set at 1.80. The statistical analysis was performed using the Wilcoxon/Kruskal-Wallis test, ***P < 0.001.

We developed the Cq ratio-based RT-LAMP method using R203M mutant-type and wild-type plasmids in triplicate experiments with different concentrations. As shown in from Figure 3A, the time gap between the R203M type and the wild type always maintained a sufficient difference in the RT-LAMP reaction with different concentrations of the template (from 10^3^ copies/mL to 10^8^ copies/mL). The mutant Cq value was divided by the conservative Cq value to obtain the final Cq ratio. The amplification templates of these two Cq values were the same type of plasmid template with the same concentration. The results and comparison for the Cq ratio of the two plasmid templates containing different alleles are shown in Figure 3B (left). A Cq ratio threshold of 1.80 was established (the threshold is the middle value of the ratio of the two groups). Then, we performed the same serial dilution and amplification tests using RNA extracted from Delta variant samples and non-Delta variant samples, and the Cq ratios are shown in Figure 3B (right). The RNA of non-Delta variants contains different types sequenced as 20A, 20I (Alpha, V1) and 20H (Beta, V2), including common gene sequence types near the R203M mutation (Supplemental Table 3). When the Cq ratio was less than 1.80, the nucleic acid template for RT-LAMP detection was judged to contain the R203M mutation; correspondingly, when the Cq ratio was greater than 1.80, the nucleic acid template should not contain the mutation. The Cq ratios of the wild-type and R203M mutation types were significantly different, which demonstrated the feasibility of this method in identifying the R203M mutation.

### Analysis of specificity and sensitivity for RT-LAMP

#### Specificity of RT-LAMP

The primer specificity was assessed with a BLAST search of the GenBank nucleotide database by aligning *N* gene sequences from different viruses. The BLAST results indicated that RT-LAMP primers for SARS-CoV-2 (Conserved and R203M primers) were specific and had a substantial nucleotide mismatch with SARS, MERS, and other viruses (Figure 2A). The specificity of the RT-LAMP assay was further verified in a cross-reactivity experiment using common viral pathogens and human-pathogenic coronaviruses (12 types). Reaction mixtures containing template SARS-CoV-2 *N* gene DNA (wild-type, R203M mutant-type, R203K/G204R mutant-type and T205I mutant-type) yielded positive results, whereas all other reaction mixtures, including a DEPC water negative control, yielded negative results (Figure 2C), supporting the specificity of this assay for SARS-CoV-2 and Delta. The same concentration of R203K/G204R and the T205I mutant *N* gene plasmids yielded higher Cq values than the wild-type under the amplification of the R203M primer set, indicating that the R203M primer set distinguishes Delta from other common variants.

#### Assessment of the sensitivity

Dilution experiments with RNA from wild-type and R203M mutant-type samples templates were performed to determine the 95% limit of detection (LOD 95) of the developed RT-LAMP assay. The calculated LOD 95 values of RT-LAMP for the Delta variant, non-Delta variant, and conserved *N* gene sequence of SARS-CoV-2 were 481 (130-831), 1279 (547-2011) cand 69 (42–96) copies/reaction, respectively (Figure 2D). In addition, when the extracted nucleic acid was as low as 15–60 copies/reaction, the two sets of primers retained detection rate of approximately 50% for the clinical sample and achieved a single reaction differentiation for the R203M mutant-containing template from the wild-type target.

### Validation of the strategy using clinical samples

**Figure 4.**
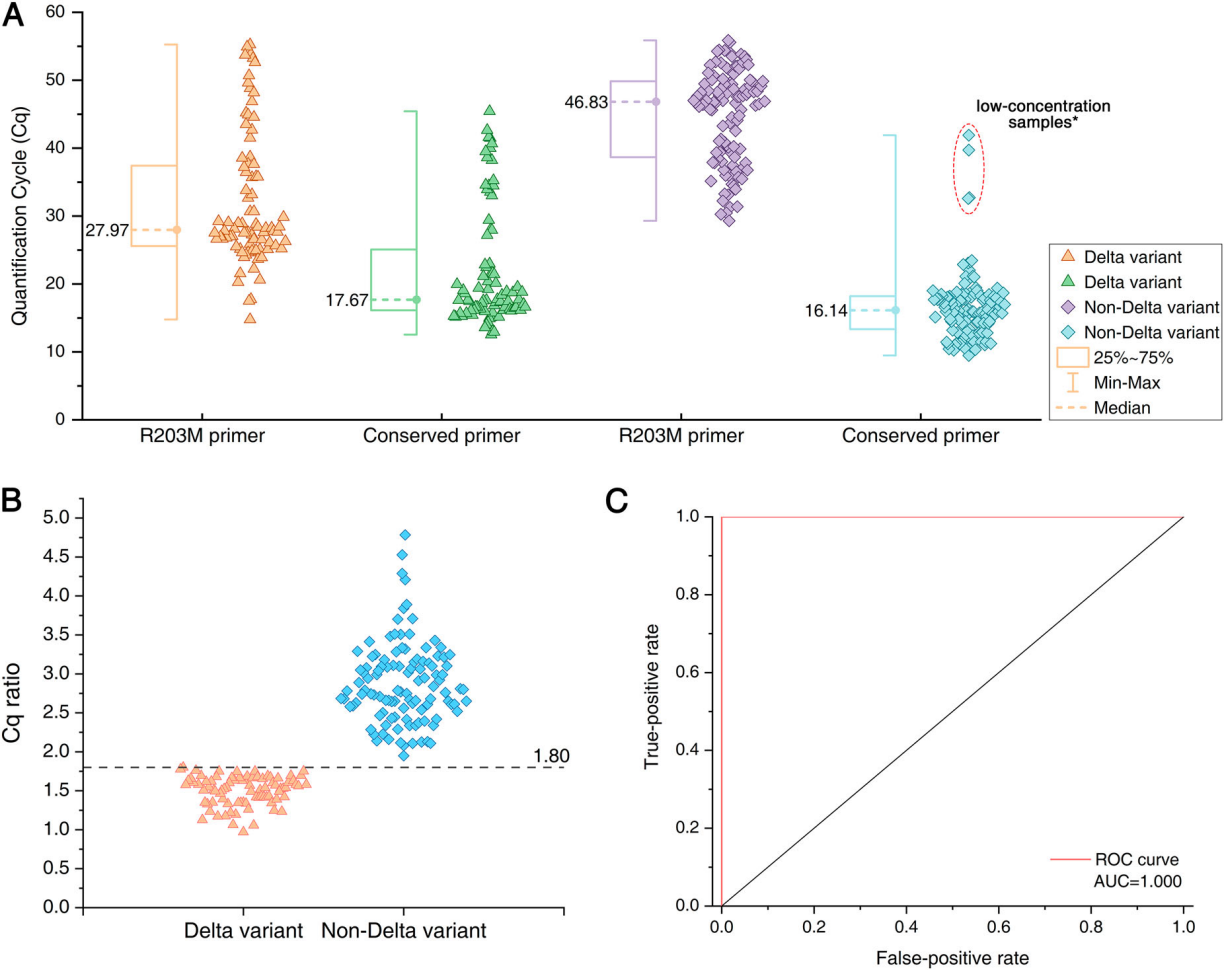
Analysis of RT-LAMP results and Delta genotyping methods from clinical samples. (A) Comparison of the Cq values for R203M and conserved primers on the amplification of clinical samples. Box plots indicate the minimum and maximum value (whiskers), median (middle dotted line), and 25th and 75th percentiles (box). *Low-concentration samples: The four low-concentration non-Delta samples circled by the red dotted line have no corresponding amplification Cq value using R203M primers. (B) Comparison of the Cq ratio between Delta and non-Delta variants. The cutoff of the Cq ratio is set at 1.80 (dotted line). The Cq ratio values are significantly different in Delta and non-Delta variants. Statistical analysis was performed using the Wilcoxon/Kruskal-Wallis test, ***P < 0.001. (C) ROC (receiver operating characteristics with area under curve) curves for Delta variant identification as measured by the developed RT-LAMP method.

We then evaluated 498 nasopharyngeal clinical samples. For comparative purposes, the samples were tested with a commercial RT-qPCR kit at the same time. All the samples were 198 SARS-CoV-2 positive and 300 SARS-CoV-2 negative in two ways. Eighty-four of 198 SARS-CoV-2-positive samples were Delta determined by sequencing (Supplemental Table 3). The clinical performance results of RT-LAMP and RT-qPCR, including the sensitivity, specificity, and other results, are shown in Table 2. The RT-LAMP assay had equally high sensitivity, specificity and accuracy (100.00%) in identifying SARS-CoV-2, compared to traditional RT-qPCR (Table 2). In the analysis of Delta detection, among 198 RT-LAMP positive samples, 84 Delta samples were correctly identified by the RT-LAMP method at the Cq ratio threshold of 1.80, and the remaining 114 samples were judged to be non-Delta variants (Table 2, Figure 4A–C).

**Table 2.** Performances of the developed RT-LAMP assay in detecting Delta and SARS-CoV-2 from clinical samples

Subjects	No. of samples		% (95% CI)				
	SARS-CoV-2 positive (n = 198)	SARS-CoV-2 negative (n = 300)	Sensitivity	Specificity	Positive predictive value	negative predictive value	Accuracy
RT-LAMP (conserved primer)			100.00 (98.15–100.00)	100.00 (98.74–100.00)	100.00 (98.15–100.00)	100.00 (98.74–100.00)	100.00 (99.23–100.00)
Positive	198	0					
Negative	0	300					
RT-LAMP (R203M primer)			97.98 (94.92–99.21)	100.00 (98.74–100.00)	100.00 (98.06–100.00)	98.68 (96.67–99.49)	99.20 (97.95–99.69)
Positive	194	0					
Negative	4	300					
RT-qPCR			100.00 (98.15–100.00)	100.00 (98.74–100.00)	100.00 (98.15–100.00)	100.00 (98.74–100.00)	100.00 (99.23–100.00)
Positive	198	0					
Negative	0	300					
	Delta (n = 84)	Non-Delta (n = 114)					
RT-LAMP (Cq ratio-based)			100.00 (95.63–100.00)	100.00 (96.74–100.00)	100.00 (95.63–100.00)	100.00 (96.74–100.00)	100.00 (98.10–100.00)
Positive	84	0					
Negative	0	114					

It is worth noting that among the 114 non-Delta-positive samples, 4 did not yield the effective Cq value of the R203M primer (the corresponding conserved primer Cq value in the red dotted circle in Figure 4A). The RT-qPCR and conserved LAMP primers amplified normally, the Cq value showed that the concentration of these samples was very low, and these 4 samples were directly judged as non-Delta. Compared with the Delta-positive samples with the same low concentration, the two sets of RT-LAMP primers have effective Cq values, and the Cq ratios also meet the threshold judgment criteria. This observation indicates that when the concentration of the unknown positive sample is close to the detection limit, the Delta sample can be judged by the ratio normally, while the non-Delta sample may not be able to amplify the R203M primer due to the sequence difference, but it also shows that the gene sequence does not match the R203M mutation. The sample can be directly judged as a non-Delta variant (when conservative primers should be amplified normally).

The performances of the RT-LAMP assay at Delta detection were as follows: the sensitivity, specificity and positive predictive value (PPV) were all 100.00%, and the negative predictive value (NPV) and accuracy were 100.00% for Delta detection (Table 2). As reported in Figure 4B, the RT-LAMP assay at a Cq ratio cutoff of 1.80 achieved superior diagnostic performance (AUC = 1.00) (Figure 4C), indicating that the rapid and inexpensive RT-LAMP assay was competent at identifying the R203M mutation in clinical samples as confirmed by sequencing.

## Discussion

Approaches based on LAMP are routinely applied for sequence identifications of pathogens rather than being common technologies for single nucleotide polymorphism (SNP) genotyping. In the present study, we first appraised the application of RT-LAMP in identifying globally dominant Delta variants based on R203M discrimination. Our results corroborate the existing evidence from other studies [12–17], showing that RT-LAMP could serve as a rapid and cost-effective screening test for SARS-CoV-2 detection and Delta recognition.

The genotyping RT-LAMP method was developed by analyzing the significant discrepancy in amplification efficiency of two sets of primers, the Delta-specific R203M primer set and the conserved *N* gene primer set, in which the R203M primer set was the core part to realize the amplification difference based on SNP discrimination. We found that the R203M primer set had a favorable capability for distinguishing mutations but a moderate amplification ability for the target sequence. Despite a relatively high LOD of RT-LAMP, the analytical sensitivity of this strategy (481 copies/reaction) is adequate to detect the R203M mutation in clinical use. Clinical sample testing of this assay further indicated a diagnostic sensitivity of 100%, a specificity of 100.00% for SARS-CoV-2, a sensitivity and specificity of 100.00% for Delta, as confirmed by sequencing, which was comparable to that of an RT-qPCR melt method for VOCs [7]. In addition, patients with Delta infections have up to approximately 1000-fold higher levels of viral RNA in their nasopharynx secretions than those with the initial SARS-CoV-2 strain per several studies [1], which is conducive to our genotyping RT-LAMP genotyping. Taken together, these observations show that our method can specifically detect and differentiate the Delta variants with high confidence in real samples.

In our study, we selected a signature mutation of the *N* gene R203M as the detective target, which accounted for 98.6% in the sequenced Delta variants. R203M is considered to be potentially destructive to the nucleocapsid protein structure according to the cluster analysis of SNP data [18]. Studies have reported that R203M could enhance the adaptability of the Delta variants and significantly bolster their infectiousness. Syed AM et al. found that the R203M mutation allowed Delta to deposit 10 times more mRNA into host cells than the wild-type virus in luciferase experiments, and they further demonstrated that R203M-mutant viruses generated 51 times more infectious virus in a live coronavirus model [23]. A study by Li et al. [2] also showed that the Delta variant (containing nucleocapsid: R203M) produced an immensely higher viral load in patients.

Delta variants account for the largest proportion of confirmed cases of SARS-CoV-2 worldwide (cumulative prevalence 51% as of Feb 26, 2022) [25,26] and are continually evolving. The Delta epidemic in Xi'an, China, in January 2022 was the greatest community spread in China since early 2020, demonstrating that Delta remains highly transmissible and virulent. Additionally, the new Omicron variant, which has generated a new round of large-scale infection in recent months, is more similar to Alpha and Lambda in the mutation of the *N* gene (i.e. the R203K/G204R mutation). The nucleic acid sequence region corresponding to the core primer LF of the R203M primer set in the Omicron variation differs significantly from that of Delta, whereas the conserved primer area has no significant alteration. In the absence of Omicron clinical samples, we can reasonably speculate that the R203M primer provides good discrimination between Omicron and Delta. The RT-LAMP method also has foreseeable prospects for Omicron variant identification or mutation detection. The appearance of the Omicron variant also implies that SARS-CoV-2 may evolve into more high-risk variants in the future, and the appearance of other variants near the R203M site is beneficial to the ability of the current R203M primer to identify Delta.

## Conclusion

We developed a highly specific and cost-effective RT-LAMP strategy to identify Delta-specific SNP mutations on a conventional detection platform within a turnaround time of approximately 1 h, increasing access to the real-time monitoring of Delta variants and SARS-CoV-2 infection.

## Supplementary Material

Supplemental Material

## References

[1] Wang Y, Chen R, Hu F, et al. Transmission, viral kinetics and clinical characteristics of the emergent SARS-CoV-2 Delta VOC in guangzhou. China. EClinicalMedicine. 2021;40:101129. 34541481 10.1016/j.eclinm.2021.101129 PMC8435265

[2] Li B, Deng A, Li K, et al. Viral infection and transmission in a large, well-traced outbreak caused by the SARS-CoV-2 Delta variant. Nat Commun. 2022;13(1):1–9. 35075154 10.1038/s41467-022-28089-y PMC8786931

[3] Iacobucci G. COVID-19: single vaccine dose is 33% effective against variant from India, data show. Br Med J. 2021 May 25;373:n1346. 34035039 10.1136/bmj.n1346

[4] Lopez Bernal J, Andrews N, Gower C, et al. Effectiveness of COVID-19 vaccines against the B.1.617.2 (Delta) variant. N Engl J Med. 2021;385(7):585–594. 34289274 10.1056/NEJMoa2108891 PMC8314739

[5] Fisman DN, Tuite AR. Evaluation of the relative virulence of novel SARS-CoV-2 variants: a retrospective cohort study in Ontario, Canada. CMAJ. 2021;93(42):E1619–E1625. 10.1503/cmaj.211248 PMC8562985 34610919

[6] Cyranoski D. Alarming COVID variants show vital role of genomic surveillance. Nature. 2021;589:337–338. 33452508 10.1038/d41586-021-00065-4

[7] Banada P, Green R, Banik S, et al. A simple reverse transcriptase PCR melting-temperature assay to rapidly screen for widely circulating SARS-CoV-2 variants. J Clin Microbiol. 2021;59(10):e0084521. 34288729 10.1128/JCM.00845-21 PMC8451443

[8] Multiplexed RT-qPCR to screen for SARS-COV-2 B.1.1.7 variants: preliminary results. [Internet] [cited 2021 October]. Available from: https://virological.org/t/multiplexed-rt-qpcr-to-screen-for-sars-cov-2-b-1-1-7-variants-preliminary-results/588.

[9] Ozsolak F, Milos PM. RNA sequencing: advances, challenges and opportunities. Nat Rev Genet. 2011;12(2):87–98. 21191423 10.1038/nrg2934 PMC3031867

[10] Notomi T, Okayama H, Masubuchi H, et al. Loop-mediated isothermal amplification of DNA. Nucleic Acids Res. 2000;28(12):e63. 10871386 10.1093/nar/28.12.e63 PMC102748

[11] Nagamine K, Hase T, Notomi T. Accelerated reaction by loop-mediated isothermal amplification using loop primers. Mol Cell Probes. 2002;16(3):223–229. 12144774 10.1006/mcpr.2002.0415

[12] Lalli MA, Langmade JS, Chen X, et al. Rapid and extraction-free detection of SARS-CoV-2 from saliva by colorimetric reverse-transcription loop-mediated isothermal amplification. Clin Chem. 2021;67(2):415–424. 33098427 10.1093/clinchem/hvaa267 PMC7665435

[13] Yu L, Wu S, Hao X, et al. Rapid detection of COVID-19 coronavirus using a reverse transcriptional loop-mediated isothermal amplification (RT-LAMP) diagnostic platform [letter]. Clin Chem. 2020;66:975–977. 32315390 10.1093/clinchem/hvaa102 PMC7188121

[14] Yan C, Cui J, Huang L, et al. Rapid and visual detection of 2019 novel coronavirus (SARS-CoV-2) by a reverse transcription loop-mediated isothermal amplification assay. Clin Microbiol Infect. 2020;26(6):773–779. 32276116 10.1016/j.cmi.2020.04.001 PMC7144850

[15] Kline EC, Panpradist N, Hull IT, et al. Multiplex target-redundant RT-LAMP for robust detection of SARS-CoV-2 using fluorescent universal displacement probes. medRxiv [preprint]. 2021. 08.13.21261995. Available from: https://www.medrxiv.org/content/10.1101/2021.08.13.21261995v1 10.1128/spectrum.01583-21 PMC9430505 35708340

[16] Hu X, Deng Q, Li J, et al. Development and clinical application of a rapid and sensitive loop-mediated isothermal amplification test for SARS-CoV-2 infection. mSphere. 2020;5(4):e00808–20. 32848011 10.1128/mSphere.00808-20 PMC7449630

[17] Li J, Hu X, Wang X, et al. A novel one-pot rapid diagnostic technology for COVID-19. Anal Chim Acta. 2021;1154:338310. 33736798 10.1016/j.aca.2021.338310 PMC7877206

[18] Behrmann O, Bachmann I, Spiegel M, et al. Rapid detection of SARS-CoV-2 by low volume real-time single tube reverse transcription recombinase polymerase amplification using an exo probe with an internally linked quencher (exo-IQ). Clin Chem. 2020;66(8):1047–1054. 32384153 10.1093/clinchem/hvaa116 PMC7239256

[19] Qian J, Boswell S A, Chidley C, et al. An enhanced isothermal amplification assay for viral detection. Nat Commun. 2020;11(1):1–10. 33219228 10.1038/s41467-020-19258-y PMC7679446

[20] Piepenburg O, Williams CH, Stemple DL, et al. DNA detection using recombination proteins. PLoS Biol. 2006;4(7):e204. 16756388 10.1371/journal.pbio.0040204 PMC1475771

[21] Ding S, Chen R, Chen G, et al. One-step colorimetric genotyping of single nucleotide polymorphism using probe-enhanced loop-mediated isothermal amplification (PE-LAMP). Theranostics. 2019;9(13):3723–3731. 31281509 10.7150/thno.33980 PMC6587344

[22] SARS-CoV-2 Variant Classifications and Definitions. [Internet] Center for Disease Control and Prevention; [cited 2021 October]. Available from: https://www.cdc.gov/coronavirus/2019-ncov/variants/variant-info.html.

[23] Syed AM, Taha TY, Tabata T, et al. Rapid assessment of SARS-CoV-2–evolved variants using virus-like particles. Science. 2021;374(6575):1626–1632. 34735219 10.1126/science.abl6184 PMC9005165

[24] Ma Y, Zhang B, Wang M, et al. Enhancement of polymerase activity of the large fragment in DNA polymerase I from geobacillus stearothermophilus by site-directed mutagenesis at the active site. Biomed Res Int. 2016;2016:2906484. 27981047 10.1155/2016/2906484 PMC5131239

[25] Delta Variant Report. [Internet] SARS-CoV-2 (hCoV-19) Mutation Reports Lineage | Mutation Tracker. [cited 2022 Feb]. Available from: https://outbreak.info/situation-reports/delta?loc=IND&loc=GBR&loc=USA&selected.

[26] Omicron Variant Report. [Internet] SARS-CoV-2 (hCoV-19) Mutation Reports Lineage | Mutation Tracker. [cited 2022 Feb]. Available from: https://outbreak.info/situation-reports/omicron?loc=ZAF&loc=GBR&loc=USA&selected=ZAF.

