# Supplementary material for: RT-LAMP assay for rapid detection of the R203M mutation in SARS-CoV-2 Delta variant
Source: Emerg Microbes Infect. 2022 Mar 30;11(1):978–87. doi: 10.1080/22221751.2022.2054368 (PMC8982466; doi:10.1080/22221751.2022.2054368)
Supplement: Supplemental Material [file TEMI_A_2054368_SM5548.docx]

**Supplemental Materials for Yang et al. RT-LAMP assay for rapid detection of the R203M mutation in SARS-CoV-2 Delta variant**

**Supplemental Table 1.** All screened RT-LAMP primers for the identification of R203M in the SARS-CoV-2 *N* gene

| R203M mutation primer set | Primer | Sequence(5’to3’) |
| --- | --- | --- |
| **PE1*^a^*** | **PE1-F3** | **ACGTAGTCGCAACAGTTC** |
|  | **PE1-B3** | **CTTAGAAGCCTCAGCAGC** |
|  | **PE1-FIP** | **AAGCAAGAGCAGCATCACCG-GAAATTCAACTCCAGGCAGC** |
|  | **PE1-BIP** | **CTGCTTGACAGATTGAACCAGC-GTGACAGTTTGGCCTTGTT** |
|  | PE1-LF1 | TCTAGCAGGAGAAGTTCCC**A*^b^***TACT |
|  | PE1-LF2 | TCTAGCAGGAGAAGTTCCC**A**TAC |
|  | PE1-LF3 | TTCTAGCAGGAGAAGTTCCC**A**T |
|  | **PE1-LF4** | **CATTCTAGCAGGAGAAGTTCCCA** |
|  |  |  |
| PE2 | PE2-F3 | CCTCATCACGTAGTCGCA |
|  | PE2-B3 | TGGCCTTGTTGTTGTTGG |
|  | PE2-FIP | CCATTGCCAGCCATTCTAGCA-GTTCAAGAAATTCAACTCCAG |
|  | PE2-BIP | GGTGATGCTGCTCTTGCTTT-ACCAGACATTTTGCTCTCAA |
|  | PE2-LF1 | AGAAGTTCCC**A**TACTGCTGC |
|  | PE2-LF2 | GAGAAGTTCCC**A**TACTGCTGC |
|  | PE2-LF3 | GGAGAAGTTCCC**A**TACTGCTGC |
|  | PE2-LF4 | GGAGAAGTTCCC**A**TACTGCTG |
|  |  |  |
| PE3 | PE3-F3 | AAGCCTCTTCTCGTTCCTC |
|  | PE3-B3 | AGAAGCCTCAGCAGCAGAT |
|  | PE3-FIP | GCCAGCCATTCTAGCAGGAG-ATCACGTAGTCGCAACAGT |
|  | PE3-BIP | TGCTTGACAGATTGAACCAGCT-AGTGACAGTTTGGCCTTGTT |
|  | PE3-LF1 | TTCCC**A**TACTGCTGCCTGGAG |
|  | PE3-LF2 | GTTCCC**A**TACTGCTGCCTGGA |
|  | PE3-LF3 | AGTTCCC**A**TACTGCTGCCTG |
|  | PE3-LF4 | AAGTTCCC**A**TACTGCTGCCT |
|  |  | |
| PE4 | PE4-F3 | GATCACATTGGCACCCGC |
|  | PE4-B3 | AGCAAGAGCAGCATCACC |
|  | PE4-FIP | TCCCTTCTGCGTAGAAGCCTTT-GCTAACAATGCTGCAATCGT |
|  | PE4-BIP | TCACGTAGTCGCAACAGTTCAA-CAGCCATTCTAGCAGGAGA |
|  | PE4-LB1 | AACTCCAGGCAGCAGTA**T**GGG |
|  | PE4-LB2 | ACTCCAGGCAGCAGTA**T**GG |
|  | PE4-LB3 | TCCAGGCAGCAGTA**T**GGGA |
|  | PE4-LB4 | TCCAGGCAGCAGTA**T**GGGAA |
|  |  |  |
| PE5 | PE5-F3 | ACCCGCAATCCTGCTAAC |
|  | PE5-B3 | GCAAGAGCAGCATCACCG |
|  | PE5-FIP | CCTCTGCTCCCTTCTGCGTA-AATGCTGCAATCGTGCTACA |
|  | PE5-BIP | TCACGTAGTCGCAACAGTTCAA-CATTGCCAGCCATTCTAGC |
|  | PE5-LB1 | CAGGCAGCAGTA**T**GGGAACTT |
|  | PE5-LB2 | GGCAGCAGTA**T**GGGAACTTC |
|  | PE5-LB3 | GCAGCAGTA**T**GGGAACTTCTCC |
|  | PE5-LB4 | CAGCAGTA**T**GGGAACTTCTCCT |
|  |  |  |
| PE6 | PE6-F3 | TGCTACAACTTCCTCAAGGA |
|  | PE6-B3 | AGCAAAGCAAGAGCAGCA |
|  | PE6-FIP | ATGAGGAACGAGAAGAGGCTTG-GCCAAAAGGCTTCTACGC |
|  | PE6-BIP | CGTAGTCGCAACAGTTCAAGAA-CACCGCCATTGCCAGCCATTC |
|  | PE6-LB1 | GGCAGCAGTA**T**GGGAACTT |
|  | PE6-LB2 | GGCAGCAGTA**T**GGGAACTTCT |
|  | PE6-LB3 | CAGCAGTA**T**GGGAACTTCTCCTG |
|  | PE6-LB4 | GCAGTA**T**GGGAACTTCTCCTG |

*a**: PE1 primer set marked in red was the best amplification efficiency primers (including F3/B3/FIP/BIP/LF), and was finally selected as the R203M primer set.

*b*:* R203M mutation nucleobases were marked with a yellow background in LF or LB primers which the is different from the wild type. The site nucleobase matches the T in the Delta N gene (G in the wild-type).

**Supplemental Table 2.** RT-LAMP primers for the conserved region in the SARS-CoV-2 *N* gene

| conserved region primer set | Primer | Sequence(5’to3’) |
| --- | --- | --- |
| **A1*^a^*** | **A1-F3** | **ACCGAAGAGCTACCAGACG** |
|  | **A1-B3** | **GCATTGTTAGCAGGATTGCG** |
|  | **A1-FIP** | **TCTGGCCCAGTTCCTAGGTAGT-AATTCGTGGTGGTGACGG** |
|  | **A1-BIP** | **AGCTGGACTTCCCTATGGTGCT-GGTGTATTCAAGGCTCCCTC** |
|  | **A1-LF** | **ACCATCTTGGACTGAGATCTTTCA** |
|  | **A1-LB** | **GACGGCATCATATGGGTTGCA** |
|  |  |  |
| A2 | A2-F3 | ACTACCTAGGAACTGGGCC |
|  | A2-B3 | GAAGAGGCTTGACTGCCG |
|  | A2-FIP | GGCTCCCTCAGTTGCAACCC-GAAGCTGGACTTCCCTATGG |
|  | A2-BIP | GCACCCGCAATCCTGCTAACA-TGCGTAGAAGCCTTTTGGC |
|  | A2-LF | TGATGCCGTCTTTGTTAGCA |
|  | A2-LB | GCTACAACTTCCTCAAGGAACA |
|  |  |  |
| A3 | A3-F3 | ACCGAAGAGCTACCAGACG |
|  | A3-B3 | TGCAGCATTGTTAGCAGGAT |
|  | A3-FIP | TCTGGCCCAGTTCCTAGGTAGT-TCGTGGTGGTGACGGTAA |
|  | A3-BIP | AGACGGCATCATATGGGTTGCA-CGGGTGCCAATGTGATCT |
|  | A3-LF | ACCATCTTGGACTGAGATCTTTCA |
|  | A3-LB | CTGAGGGAGCCTTGAATACACC |
|  |  |  |
| A4 | A4-F3 | TGGCTACTACCGAAGAGCT |
|  | A4-B3 | TGCAGCATTGTTAGCAGGAT |
|  | A4-FIP | TCTGGCCCAGTTCCTAGGTAGT-CCAGACGAATTCGTGGTGG |
|  | A4-BIP | AGACGGCATCATATGGGTTGCA-CGGGTGCCAATGTGATCT |
|  | A4-LF | ACCATCTTGGACTGAGATCTTTCA |
|  | A4-LB | GAGGGAGCCTTGAATACACCAA |
|  |  |  |
| B1 | B1-F3 | TCGGCAAAAACGTACTGC |
|  | B1-B3 | GTGACTTCCATGCCAATG |
|  | B1-FIP | CCTTGGGTTTGTTCTGGACC-CACTAAAGCATACAATGTAACACA |
|  | B1-BIP | CAGGAACTAATCAGACAAGGAACTG-TTCCGAAGAACGCTGAAG |
|  | B1-LF | ACGTCTGCCGAAAGCT |
|  | B1-LB | TTGGCCGCAAATTGCACAA |
|  |  |  |
| B2 | B2-F3 | AACACAAGCTTTCGGCAG |
|  | B2-B3 | CATCCAATTTGATGGCACC |
|  | B2-FIP | TGCGGCCAATGTTTGTAATCAG-CCAAGGAAATTTTGGGGAC |
|  | B2-BIP | CGCTTCAGCGTTCTTCGGAA-GTAGGTCAACCACGTTCC |
|  | B2-LF | TCCTTGTCTGATTAGTTCCTG |
|  | B2-LB | TGTCGCGCATTGGCATGG |
|  |  |  |
| B3 | B3-F3 | ACCAGGAACTAATCAGACAAG |
|  | B3-B3 | GACTTGATCTTTGAAATTTGGATCT |
|  | B3-FIP | TTCCGAAGAACGCTGAAGCG-GAACTGATTACAAACATTGGCC |
|  | B3-BIP | CGCATTGGCATGGAAGTCAC-ATCCAATTTGATGGCACCT |
|  | B3-LF | CTGGGGGCAAATTGTGCAAT |
|  | B3-LB | TCGGGAACGTGGTTGACCTA |
|  |  |  |
|  |  |  |
| B4 | B4-F3 | GGCAAAAACGTACTGCCACT |
|  | B4-B3 | GCACCTGTGTAGGTCAACC |
|  | B4-FIP | TCTGATTAGTTCCTGGTCCCCA-ACAAGCTTTCGGCAGACG |
|  | B4-BIP | GCCGCAAATTGCACAATTTGC-GGTGTGACTTCCATGCCAA |
|  | B4-LF | TTCCTTGGGTTTGTTCTGGAC |
|  | B4-LB | CGCTTCAGCGTTCTTCGGAAT |

*a*: A1 primer set marked in red was the best amplification efficiency primers (including F3/B3/FIP/BIP/LF/LB), and was finally selected as the conserved primer set.

**Supplemental Table 3.** Results of RT-LAMP and RT-qPCR assays on positive clinical samples.

| **ID of Positive samples** | **Sequencing results** | **Cq value of RT-qPCR*** | **Cq value of RT-LAMP**  **(R203M region)** | **Cq value of RT-LAMP**  **(conserved region)** | **Cq ratio**  **(R203M Cq/ conserved region Cq)** |
| --- | --- | --- | --- | --- | --- |
| 2 | 20H (Beta, V2) | 8.88 | 35.65 | 13.34 | 2.67 |
| 7 | 20I(Alpha,V1) | 5.25 | 40.11 | 10.32 | 3.89 |
| 11 | 20H(Beta,V2) | 7.31 | 32.23 | 12.81 | 2.52 |
| 38 | 20H(Beta,V2) | 11.85 | 40.23 | 17.18 | 2.34 |
| 47 | 21A (Delta) | 8.26 | 20.61 | 13.04 | 1.58 |
| 48 | 20A | 6.57 | 34.69 | 13.02 | 2.66 |
| 64 | 20I(Alpha,V1) | 5.83 | 37.84 | 11.02 | 3.43 |
| 80 | 20A | 6.48 | 30.27 | 11.44 | 2.65 |
| 84 | 20B | 6.24 | 36.76 | 11.19 | 3.29 |
| 88 | 21A (Delta) | 15.17 | 30.68 | 17.56 | 1.75 |
| 93 | 20H(Beta,V2) | 7.27 | 38.50 | 12.65 | 3.04 |
| 96 | 21A (Delta) | 13.30 | 27.65 | 15.72 | 1.76 |
| 111 | 20J(Gamma,V3) | 13.30 | 37.91 | 17.78 | 2.13 |
| 119 | 20A | 5.90 | 31.26 | 11.78 | 2.65 |
| 127 | 20H(Beta,V2) | 6.06 | 35.97 | 11.72 | 3.07 |
| 135 | 20I(Alpha,V1) | 5.91 | 39.40 | 11.21 | 3.51 |
| 139 | 20I(Alpha,V1) | 6.96 | 29.30 | 12.08 | 2.43 |
| 149 | 21A (Delta) | 8.23 | 20.26 | 13.58 | 1.49 |
| 150 | 20I(Alpha,V1) | 5.01 | 39.35 | 11.22 | 3.51 |
| 151 | 20I(Alpha,V1) | 5.72 | 40.85 | 11.01 | 3.71 |
| 154 | 20A | 7.78 | 31.38 | 11.22 | 2.80 |
| 158 | 20H (Beta, V2) | 9.85 | 39.60 | 15.46 | 2.56 |
| 187 | 20H(Beta,V2) | 6.10 | 35.73 | 12.82 | 2.79 |
| 194 | 20A | 13.11 | 34.45 | 15.94 | 2.16 |
| 218 | 21C(Epsilon) | 6.46 | 33.26 | 11.49 | 2.89 |
| 220 | 20I(Alpha,V1） | 5.78 | 36.36 | 9.48 | 3.84 |
| 222 | 21A (Delta) | 7.09 | 17.50 | 12.93 | 1.35 |
| 224 | 21A (Delta) | 13.46 | 24.63 | 16.07 | 1.53 |
| 228 | 21A (Delta) | 16.44 | 26.62 | 19.95 | 1.33 |
| 235 | 21D(Eta) | 12.18 | 41.04 | 16.98 | 2.42 |
| 241 | 21C(Epsilon) | 12.77 | 35.15 | 16.66 | 2.11 |
| 244 | 20A | 17.58 | 43.68 | 19.04 | 2.29 |
| 251 | 21A (Delta) | 6.89 | 17.75 | 12.53 | 1.42 |
| 252 | 21A (Delta) | 11.09 | 24.28 | 15.43 | 1.57 |
| 254 | 21A (Delta) | 16.75 | 26.80 | 18.73 | 1.43 |
| 260 | 20I(Alpha,V1) | 6.41 | 36.53 | 11.48 | 3.18 |
| 261 | 20A | 7.72 | 33.94 | 12.90 | 2.63 |
| 266 | 21A (Delta) | 7.20 | 14.76 | 15.25 | 0.97 |
| 275 | 20B | 9.99 | 40.47 | 14.75 | 2.74 |
| 278 | 20H(Beta,V2) | 5.48 | 37.23 | 11.52 | 3.23 |
| 288 | 20I(Alpha,V1) | 8.27 | 39.90 | 12.86 | 3.10 |
| 290 | 21A (Delta) | 16.29 | 28.97 | 19.42 | 1.49 |
| 305 | 20A | 6.51 | 30.45 | 11.46 | 2.66 |
| 306 | 20C | 13.16 | 37.84 | 17.69 | 2.14 |
| 310 | 21C(Epsilon) | 11.29 | 33.13 | 15.96 | 2.08 |
| 315 | 21A (Delta) | 17.86 | 25.88 | 19.24 | 1.35 |
| 320 | 21A (Delta) | 24.65 | 38.56 | 27.20 | 1.42 |
| 327 | 21A (Delta) | 14.78 | 25.09 | 16.61 | 1.51 |
| 338 | 21A (Delta) | 24.03 | 37.63 | 22.46 | 1.68 |
| 346 | 20I(Alpha,V1) | 4.51 | 36.80 | 10.57 | 3.48 |
| 351 | 21C(Epsilon) | 25.18 | - | 41.90 | - |
| 352 | 21A (Delta) | 13.28 | 25.15 | 16.34 | 1.54 |
| 353 | 20H(Beta,V2) | 21.30 | - | 39.71 | - |
| 354 | 20C | 18.29 | 52.56 | 17.03 | 3.09 |
| 355 | 20I(Alpha,V1) | 10.51 | 52.10 | 17.01 | 3.06 |
| 356 | 21A (Delta) | 11.28 | 23.90 | 15.15 | 1.58 |
| 357 | 20I(Alpha,V1) | 8.09 | 49.19 | 15.67 | 3.14 |
| 358 | 20A | 12.30 | 49.62 | 18.05 | 2.75 |
| 359 | 20I(Alpha,V1) | 7.53 | 53.50 | 16.04 | 3.34 |
| 360 | 21A (Delta) | 9.79 | 29.84 | 17.04 | 1.75 |
| 361 | 20A | 8.79 | 46.27 | 14.25 | 3.25 |
| 362 | 21A (Delta) | 14.21 | 35.82 | 21.13 | 1.70 |
| 363 | 20I(Alpha,V1) | 7.94 | 49.12 | 15.96 | 3.08 |
| 364 | 20I(Alpha,V1) | 8.07 | 48.00 | 15.91 | 3.02 |
| 365 | 20B | 15.16 | 47.78 | 18.82 | 2.54 |
| 366 | 21A (Delta) | 23.14 | 44.92 | 38.22 | 1.18 |
| 367 | 21A (Delta) | 15.80 | 38.72 | 22.97 | 1.69 |
| 368 | 20I(Alpha,V1) | 11.32 | 54.37 | 17.55 | 3.10 |
| 369 | 21A (Delta) | 13.11 | 23.66 | 15.70 | 1.51 |
| 370 | 20I(Alpha,V1) | 9.99 | 49.85 | 17.93 | 2.78 |
| 371 | 20I(Alpha,V1) | 12.79 | 50.05 | 18.68 | 2.68 |
| 372 | 20A | 10.03 | 47.88 | 17.54 | 2.73 |
| 373 | 20I(Alpha,V1) | 4.41 | 38.01 | 14.18 | 2.68 |
| 374 | 20I(Alpha,V1) | 9.17 | 41.33 | 17.68 | 2.34 |
| 375 | 21A (Delta) | 9.92 | 28.43 | 17.71 | 1.61 |
| 376 | 21A (Delta) | 9.66 | 26.63 | 16.59 | 1.61 |
| 377 | 20I(Alpha,V1) | 5.04 | 43.25 | 14.18 | 3.05 |
| 378 | 21A (Delta) | 18.86 | 41.52 | 29.40 | 1.41 |
| 379 | 20I(Alpha,V1) | 12.22 | 50.92 | 17.49 | 2.91 |
| 380 | 20I(Alpha,V1) | 7.78 | 38.67 | 15.81 | 2.45 |
| 381 | 21A (Delta) | 11.12 | 29.05 | 17.47 | 1.66 |
| 382 | 20H(Beta,V2) | 9.72 | 53.04 | 15.54 | 3.41 |
| 383 | 21A (Delta) | 17.46 | 33.81 | 21.42 | 1.58 |
| 384 | 20H(Beta,V2) | 5.47 | 51.86 | 10.84 | 4.78 |
| 385 | 21A (Delta) | 18.01 | 32.68 | 21.62 | 1.51 |
| 386 | 20I(Alpha,V1) | 9.28 | 52.70 | 17.62 | 2.99 |
| 387 | 20H(Beta,V2) | 18.02 | 49.84 | 23.45 | 2.13 |
| 388 | 21A (Delta) | 27.17 | 53.27 | 45.43 | 1.17 |
| 389 | 20B | 18.36 | 49.48 | 20.08 | 2.46 |
| 390 | 20I(Alpha,V1) | 11.56 | 53.50 | 17.30 | 3.09 |
| 391 | 20A | 5.85 | 44.77 | 10.44 | 4.29 |
| 392 | 21A (Delta) | 10.77 | 24.62 | 15.05 | 1.64 |
| 393 | 21A (Delta) | 13.10 | 27.27 | 20.38 | 1.34 |
| 394 | 20I(Alpha,V1) | 8.63 | 48.25 | 16.36 | 2.95 |
| 395 | 21A (Delta) | 22.98 | 53.71 | 38.62 | 1.39 |
| 396 | 21C(Epsilon) | 14.87 | 55.87 | 15.09 | 3.70 |
| 397 | 21A (Delta) | 13.25 | 27.59 | 17.61 | 1.57 |
| 398 | 21A (Delta) | 10.75 | 24.64 | 15.10 | 1.63 |
| 399 | 20I(Alpha,V1) | 7.55 | 50.18 | 15.73 | 3.19 |
| 400 | 20I(Alpha,V1) | 5.96 | 47.00 | 14.48 | 3.25 |
| 401 | 20I(Alpha,V1) | 10.74 | 52.28 | 17.33 | 3.02 |
| 402 | 21A (Delta) | 7.29 | 21.55 | 14.00 | 1.54 |
| 403 | 21A (Delta) | 13.57 | 36.46 | 34.49 | 1.06 |
| 404 | 21A (Delta) | 15.15 | 27.60 | 18.37 | 1.50 |
| 405 | 21A (Delta) | 9.58 | 22.20 | 14.35 | 1.55 |
| 406 | 21A (Delta) | 13.56 | 26.95 | 21.61 | 1.25 |
| 407 | 21A (Delta) | 13.37 | 25.64 | 19.09 | 1.34 |
| 408 | 21A (Delta) | 20.03 | 45.21 | 33.92 | 1.33 |
| 409 | 20I(Alpha,V1) | 8.17 | 54.04 | 16.28 | 3.32 |
| 410 | 21A (Delta) | 22.35 | 44.58 | 39.52 | 1.13 |
| 411 | 21A (Delta) | 14.59 | 28.25 | 17.46 | 1.62 |
| 412 | 20A | 8.92 | 42.53 | 13.19 | 3.22 |
| 413 | 21A (Delta) | 14.45 | 37.21 | 22.85 | 1.63 |
| 414 | 21C(Epsilon) | 19.49 | 54.45 | 23.29 | 2.34 |
| 415 | 20I(Alpha,V1) | 4.84 | 47.54 | 14.48 | 3.28 |
| 416 | 21A (Delta) | 19.41 | 26.97 | 18.89 | 1.43 |
| 417 | 21A (Delta) | 26.71 | 49.63 | 41.49 | 1.20 |
| 418 | 21A (Delta) | 24.50 | 50.69 | 41.61 | 1.22 |
| 419 | 21A (Delta) | 9.73 | 27.05 | 16.15 | 1.67 |
| 420 | 21C(Epsilon) | 19.03 | 52.24 | 22.86 | 2.29 |
| 421 | 21A (Delta) | 9.51 | 27.52 | 15.28 | 1.80 |
| 422 | 20I(Alpha,V1) | 14.38 | 48.68 | 18.41 | 2.64 |
| 423 | 20C | 21.95 | 50.67 | 21.16 | 2.39 |
| 424 | 20A | 17.42 | 46.63 | 22.04 | 2.12 |
| 425 | 21A (Delta) | 13.36 | 27.78 | 18.59 | 1.49 |
| 426 | 20A | 16.06 | 47.02 | 21.11 | 2.23 |
| 427 | 21A (Delta) | 22.06 | 46.88 | 33.03 | 1.42 |
| 428 | 20I(Alpha,V1) | 17.11 | 50.04 | 19.39 | 2.58 |
| 429 | 20J(Gamma,V3) | 16.61 | 53.82 | 18.02 | 2.99 |
| 430 | 20I(Alpha,V1) | 9.96 | 52.67 | 18.03 | 2.92 |
| 431 | 21A (Delta) | 12.90 | 33.17 | 19.57 | 1.69 |
| 432 | 20A | 13.65 | 48.44 | 20.42 | 2.37 |
| 433 | 21A (Delta) | 10.09 | 26.59 | 15.98 | 1.66 |
| 434 | 20I(Alpha,V1) | 11.62 | 46.13 | 17.66 | 2.61 |
| 435 | 21A (Delta) | 16.37 | 35.78 | 20.14 | 1.78 |
| 436 | 20A | 24.74 | - | 32.58 | - |
| 437 | 20A | 16.07 | 46.72 | 21.02 | 2.22 |
| 438 | 21A (Delta) | 9.47 | 25.21 | 16.14 | 1.56 |
| 439 | 20A | 6.62 | 48.37 | 14.48 | 3.34 |
| 440 | 20I(Alpha,V1) | 10.53 | 35.46 | 18.20 | 1.95 |
| 441 | 20H(Beta,V2) | 8.49 | 48.21 | 14.45 | 3.34 |
| 442 | 20I(Alpha,V1) | 12.73 | 48.20 | 18.48 | 2.61 |
| 443 | 21C(Epsilon) | 23.95 | - | 32.72 | - |
| 444 | 20A | 11.96 | 49.33 | 17.70 | 2.79 |
| 445 | 21A (Delta) | 10.86 | 28.11 | 16.63 | 1.69 |
| 446 | 21A (Delta) | 9.42 | 29.23 | 17.62 | 1.66 |
| 447 | 20I(Alpha,V1) | 7.82 | 37.45 | 16.03 | 2.34 |
| 448 | 21A (Delta) | 13.44 | 28.40 | 17.74 | 1.60 |
| 449 | 21A (Delta) | 11.28 | 24.85 | 14.92 | 1.67 |
| 450 | 21A (Delta) | 21.30 | 53.88 | 39.97 | 1.35 |
| 451 | 20I(Alpha,V1) | 7.51 | 44.10 | 15.70 | 2.81 |
| 452 | 21A (Delta) | 10.53 | 23.91 | 16.33 | 1.46 |
| 453 | 21A (Delta) | 13.12 | 26.10 | 16.10 | 1.62 |
| 454 | 20I(Alpha,V1) | 8.49 | 45.72 | 16.56 | 2.76 |
| 455 | 20I(Alpha,V1) | 11.28 | 51.68 | 18.20 | 2.84 |
| 456 | 20A | 12.34 | 46.98 | 19.46 | 2.41 |
| 457 | 20A | 5.83 | 46.41 | 10.25 | 4.53 |
| 458 | 21A (Delta) | 25.84 | 48.82 | 38.70 | 1.26 |
| 459 | 21A (Delta) | 12.40 | 25.52 | 16.60 | 1.54 |
| 460 | 21A (Delta) | 10.25 | 28.22 | 16.13 | 1.75 |
| 461 | 21A (Delta) | 15.31 | 26.54 | 17.40 | 1.53 |
| 462 | 21A (Delta) | 10.96 | 27.82 | 16.43 | 1.69 |
| 463 | 21A (Delta) | 11.00 | 27.52 | 16.84 | 1.63 |
| 464 | 21A (Delta) | 11.55 | 35.68 | 33.54 | 1.06 |
| 465 | 21A (Delta) | 13.24 | 24.95 | 17.63 | 1.42 |
| 466 | 20J(Gamma,V3) | 15.86 | 47.64 | 14.82 | 3.21 |
| 467 | 21A (Delta) | 19.50 | 43.52 | 35.26 | 1.23 |
| 468 | 21C(Epsilon) | 14.87 | 55.58 | 15.85 | 3.51 |
| 469 | 21A (Delta) | 20.66 | 52.62 | 42.64 | 1.23 |
| 470 | 21A (Delta) | 18.87 | 37.67 | 27.99 | 1.35 |
| 471 | 21A (Delta) | 10.00 | 26.28 | 16.62 | 1.58 |
| 472 | 21A (Delta) | 9.33 | 27.52 | 15.78 | 1.74 |
| 473 | 20I(Alpha,V1) | 5.17 | 45.60 | 14.43 | 3.16 |
| 474 | 20I(Alpha,V1) | 6.32 | 46.90 | 15.12 | 3.10 |
| 475 | 21A (Delta) | 10.63 | 28.82 | 17.29 | 1.67 |
| 476 | 20A | 8.59 | 48.71 | 15.46 | 3.15 |
| 477 | 20A | 12.68 | 45.66 | 18.25 | 2.50 |
| 478 | 20I(Alpha,V1) | 14.89 | 49.42 | 18.59 | 2.66 |
| 479 | 20I(Alpha,V1) | 13.90 | 46.63 | 17.87 | 2.61 |
| 480 | 20I(Alpha,V1) | 14.36 | 54.27 | 18.44 | 2.94 |
| 481 | 20I(Alpha,V1) | 8.59 | 44.53 | 16.23 | 2.74 |
| 482 | 21A (Delta) | 18.75 | 48.19 | 34.60 | 1.39 |
| 483 | 21A (Delta) | 22.54 | 54.92 | 40.68 | 1.35 |
| 484 | 20I(Alpha,V1) | 12.72 | 46.50 | 17.96 | 2.59 |
| 485 | 20A | 12.50 | 48.64 | 23.08 | 2.11 |
| 486 | 20I(Alpha,V1) | 10.62 | 47.69 | 17.60 | 2.71 |
| 487 | 20I(Alpha,V1) | 15.35 | 51.65 | 18.65 | 2.77 |
| 488 | 21A (Delta) | 19.69 | 55.25 | 34.46 | 1.60 |
| 489 | 21A (Delta) | 12.41 | 28.90 | 17.21 | 1.68 |
| 490 | 20I(Alpha,V1) | 6.89 | 46.76 | 15.64 | 2.99 |
| 491 | 21A (Delta) | 13.54 | 30.67 | 18.81 | 1.63 |
| 492 | 20H(Beta,V2 | 13.52 | 51.07 | 19.02 | 2.69 |
| 493 | 21A (Delta) | 9.42 | 25.07 | 15.15 | 1.65 |
| 494 | 20H(Beta,V2) | 6.76 | 52.06 | 12.37 | 4.21 |
| 495 | 20A | 12.82 | 49.38 | 18.83 | 2.62 |
| 496 | 21A (Delta) | 18.77 | 42.69 | 27.95 | 1.53 |
| 497 | 20I(Alpha,V1) | 11.18 | 48.28 | 18.21 | 2.65 |
| 498 | 20I(Alpha,V1) | 7.15 | 47.58 | 15.30 | 3.11 |

* The commercial RT-qPCR kit (Da'an Gene) is a widely-used fast detection kit for SARS-CoV-2 in China. A Cq value of less than 30 is defined as a positive test based on the manufacturer’s instructions (the cutoff Cq value is empirically 35~40 for routine RT-qPCR).

**Supplemental Figure 1**


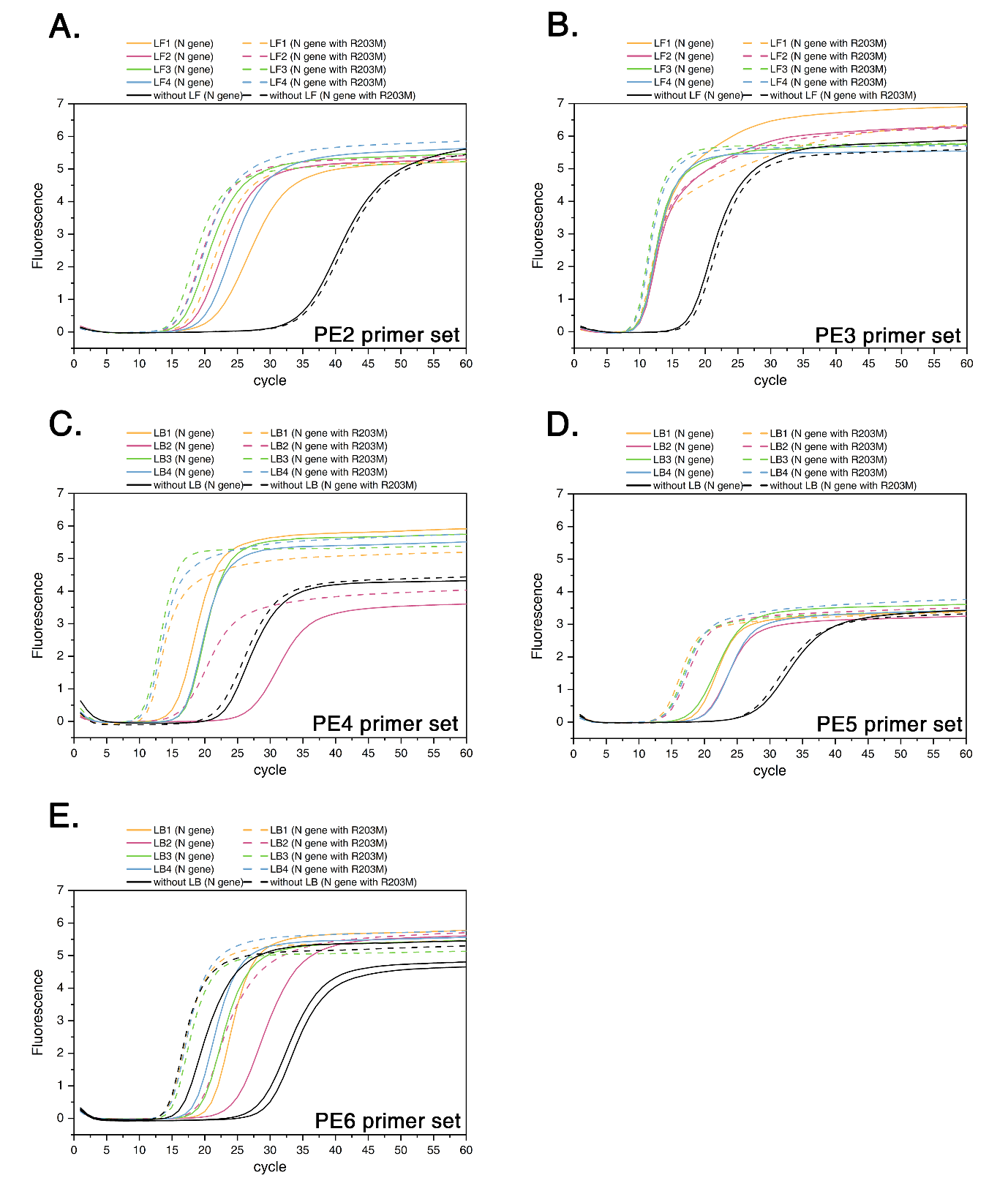


**Supplemental Figure 1:** Fluorescence amplification screening of mutant primer sets.

A, B, C, D, and E are the amplification test of LAMP primer set PE2, PE3, PE4, PE5, and PE6, respectively. The nucleic acid template concentration in all RT-LAMP reactions is 10^8^ copies/mL.

Each set of inner and outer primers (F3/B3/FIP/BIP) and its corresponding multiple loop primers were amplified and tested with wild-type *N* gene and R203M mutation type *N* gene plasmids as templates. In the figure, all the solid lines are amplification curves with the wild-type *N* gene as the template; dashed lines with the same color are amplification curves of the same set of primer pairs for the R203M mutation-type *N* gene.

**Supplemental Figure 2**


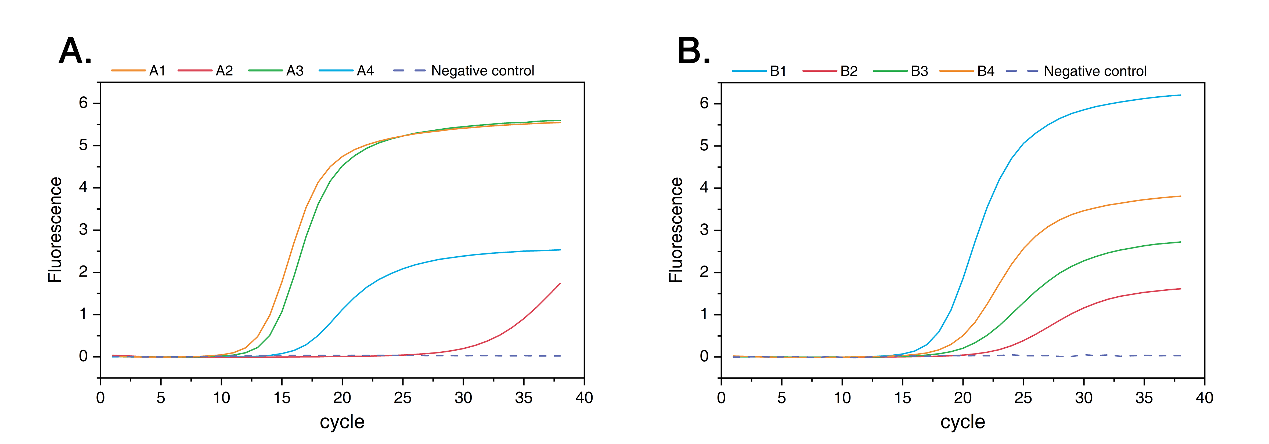


**Supplemental Figure 2:** Fluorescence amplification screening of conserved region primer sets.

A, B are the amplification test of LAMP primer set A1/A2/A3/A4 and B1/B2/B3/B4 respective. The template is 10^8^ copies/mL concentration of wild type N gene.

**Supplemental Figure 3**


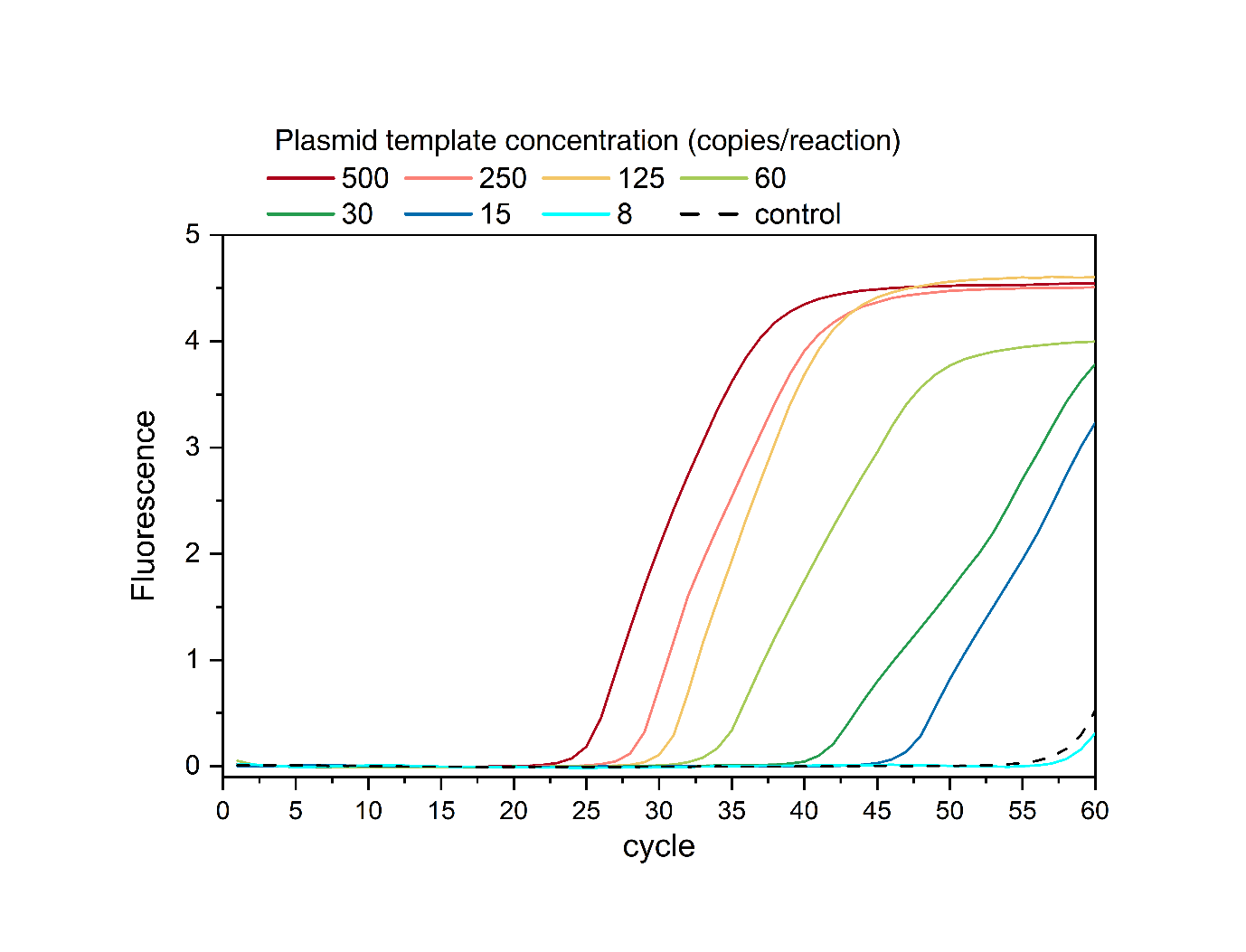


**Supplemental Figure 3：**Detection limit of the RT-LAMP assay**.**

The LOD of the RT-LAMP analysis when used as a means of detecting SARS-CoV-2 was assessed using gradient dilution plasmid templates, with DEPC water as a negative control. After 55 cycles, the control sample may have nonspecific amplification, so the detection of LOD was determined to be before 55cq.
